# Development and evaluation of a formaldehyde-stabilized tuberculin as a safe and potent alternative to phenol-based purified protein derivative for the diagnosis of animal tuberculosis

**DOI:** 10.14202/vetworld.2025.3268-3287

**Published:** 2025-10-31

**Authors:** Kairat Turgenbayev, Assiya Borsynbayeva, Amanbek Ozatbekuly, Sairan Dyusenov, Anarbek Tlepov, Rauan Turgenbayev

**Affiliations:** BioVet Scientific and Production Center, Almaty, Kazakhstan

**Keywords:** biosafety, bovine tuberculosis, diagnostic sensitivity, formaldehyde stabilization, *Mycobacterium bovis*, purified protein derivative, tuberculin, veterinary diagnostics

## Abstract

**Background and Aim:**

Bovine tuberculosis (bTB), caused by *Mycobacterium bovis*, remains a significant zoonotic threat to livestock and public health, resulting in major economic losses. The diagnostic accuracy of purified protein derivative (PPD) tuberculin, the cornerstone of *in vivo* screening, is influenced by the allergen’s composition and stabilizer. Conventional phenol-stabilized tuberculin raises toxicity and safety concerns, prompting calls for safer alternatives. This study aimed to enhance the diagnostic value of tuberculin by replacing phenol with formaldehyde as a preservative and stabilizer.

**Materials and Methods:**

Tuberculin was prepared from *M. bovis* cultures according to the Kazakhstan national standard (Standard Republic of Kazakhstan 1130) and Government standard (16739). Experimental formulations containing 0.1%–10% formaldehyde were produced, and their physicochemical, biological, and allergenic properties were assessed. Diagnostic performance was evaluated in guinea pigs sensitized with *M. bovis* and atypical mycobacteria (*Mycobacterium* *kansasii*, *Mycobacterium* *scrofulaceum*, *Mycobacterium avium*, *Mycobacterium phlei*), in calves experimentally infected with *M. bovis* Bacillus Calmette-Guérin, and in naturally infected cattle herds. Results were compared with commercial PPD tuberculin (KazBioPharm, Kazakhstan; Kursk Biofactory, Russia). Statistical analysis was performed using Student’s t-test and analysis of variance (p < 0.05).

**Results:**

Formaldehyde at 3% yielded the highest biological activity and stability without local irritation. In *M. bovis*-sensitized guinea pigs, 3% formaldehyde-stabilized tuberculin exceeded commercial PPD by 19.9% in reaction intensity. In calves, mean skin-fold thickness increased by 13.1% compared to control PPD. No cross-reactions were observed in animals sensitized to atypical mycobacteria. In a tuberculosis (TB)-affected cattle herd (n = 87), the new formulation induced a mean skin-fold thickness of 5.23 mm, approximately 10% higher than the commercial controls, and identified 21% more infected animals. Receiver Operating Characteristic (ROC) analysis confirmed superior diagnostic accuracy (area under the ROC curve = 0.928, Youden Index = 0.80).

**Conclusion:**

Replacing phenol with 3% formaldehyde significantly enhanced the sensitivity, stability, and biosafety of tuberculin without compromising specificity. The new formulation eliminates phenol toxicity while improving diagnostic yield in animal TB screening. These findings support the integration of formaldehyde-stabilized tuberculin into national and international diagnostic standards as a reliable and safer alternative for large-scale veterinary applications.

## INTRODUCTION

Of the 10 million newly reported cases of tuberculosis (TB) each year, with approximately 1.6 million deaths, an estimated 142,000 new cases and 12,500 deaths are attributed to *Mycobacterium bovis* infections [1, 2]. Cases caused by *M. bovis* in humans are categorized as “zoonotic TB,” although they are often underreported due to limited diagnostic capacity and surveillance in endemic regions [3, 4]. The World Organization for Animal Health (WOAH) recognizes *M. bovis* as the causative agent of bovine TB (bTB), a chronic zoonotic infection affecting domestic and wild animals and posing a serious challenge to global livestock industries and public health [5]. Despite notable success in eradication programs within developed nations, bTB remains prevalent in many parts of Africa, Latin America, and Asia, including India, China, and the Middle East.

According to the World Health Organization and WOAH, the global prevalence of bTB among cattle ranges from 2% to 5%, though it may exceed 10% in regions where systematic testing and culling programs are absent. *M. bovis* contributes to approximately 1.4%–2% of all human TB cases worldwide, corresponding to 140,000–200,000 new infections annually. Transmission to humans typically occurs through the consumption of unpasteurized milk and dairy products or direct contact with infected animals, posing high risks to farmers and rural communities. Economically, the disease imposes both direct and indirect losses, reduced productivity, carcass condemnation, culling, trade restrictions, and the high costs of surveillance and diagnostic programs. The Food and Agriculture Organization estimates that global annual economic losses from bTB exceed United States Dollar 3 billion. Persistent wildlife reservoirs such as badgers in the United Kingdom, deer in North America, and wild boars in Europe continue to hinder eradication efforts. These complexities underscore the urgent need for harmonized diagnostic systems and international coordination in bTB control programs [6].

The effectiveness of TB control efforts largely depends on the accuracy of diagnostic tools. The primary *in vivo* diagnostic method for bTB remains the intradermal tuberculin skin test using purified protein derivative (PPD) tuberculin [7, 8], which detects delayed-type hypersensitivity (DTH) reactions to mycobacterial antigens. Tuberculin was first developed by Robert Koch in 1890 as a “glycerin-water extract of tuberculous cultures,” later termed Koch alt tuberculin [9]. Modern PPD tuberculin is derived from mycobacterial culture filtrates, where the protein fraction is precipitated using trichloroacetic acid (TCA) and ammonium sulfate (AMS) [10–12]. The specificity of the final preparation depends on the degree of purification and removal of ballast substances [13]. Seibert first isolated biologically active crystalline tuberculin and, in 1934, developed the PPD, which became the global diagnostic standard [14, 15].

Industrial production of PPD-tuberculin in the former Soviet Union was based on the method of Govorov and Ostashko [16]. Although the diagnostic efficacy of mammalian PPD-tuberculin reaches about 80% in infected herds, para-allergic reactions are frequent, even in TB-free farms, prompting continuous improvements to enhance specificity [17–20]. WOAH currently recommends *M. bovis* strain AN-5 for international standardization of tuberculin production [21]. The diagnostic response depends on several factors, including animal age, immune competence, physiological status, and environmental conditions. Importantly, the inability to distinguish between specific and paraspecific reactions may result in the unnecessary culling of healthy, high-value livestock.

Phenol has long been used as a preservative (up to 0.3%) in tuberculin formulations since Koch’s original preparation [16]; however, it is toxic and poses safety concerns. Public dissatisfaction with phenol-containing PPD-L tuberculin has extended even to human medicine, where it contributed to growing resistance toward the Mantoux test due to its phenol content. This has fueled anti-testing sentiments in certain populations. Laskavy [22] and Laskavy *et al*. [23] demonstrated that the use of immunopharm, a preparation containing 0.1% formaldehyde, enhanced allergic responses in TB-infected animals, doubling detection rates compared with conventional tuberculin alone. The concurrent administration of immunopharm and tuberculin enhanced specific antibody formation and cellular immune response.

Formaldehyde, widely used in vaccine production as an inactivating agent, is safer and effective at very low concentrations (0.1%–0.4%) in toxoid and viral vaccines [24–26]. Replacing phenol with formaldehyde in tuberculin could therefore improve safety, enhance immunogenic stimulation, and increase public acceptance without compromising diagnostic accuracy. Accordingly, this study aimed to increase the diagnostic value of tuberculin by formulating a formaldehyde-stabilized variant as a safe alternative to phenol-based preparations.

Kazakhstan, with a cattle population exceeding 9.5 million head, conducts annual tuberculin testing as part of its national TB control program. However, in recent years, many farms have relied on non-albumin (crude) tuberculin, which lacks the purification level of PPD, reducing specificity and increasing false-positive reactions [27–29]. Consequently, many animals have shown unexplained tuberculin reactivity, often unconfirmed upon postmortem examination. These inconsistencies highlight the urgent need to refine diagnostic protocols and develop safer, more reliable allergenic preparations for accurate TB detection in animals [30–33].

Although PPD tuberculin has served as the cornerstone of bTB diagnosis for decades, its diagnostic precision and biosafety remain constrained by chemical and immunological limitations. The continued use of phenol as a preservative, despite its known toxicity, poses substantial drawbacks, including local irritation, allergen instability, and safety concerns that restrict its widespread use in both veterinary and human applications. Moreover, phenol’s potential to alter protein epitopes during storage can reduce tuberculin potency and reproducibility between batches. In field conditions, this often translates into inconsistent sensitivity and specificity, resulting in false-positive or false-negative outcomes. These inconsistencies are further compounded by the presence of atypical mycobacteria that may trigger paratuberculin reactions, particularly in regions where environmental mycobacteria are prevalent.

Previous attempts to enhance the diagnostic accuracy of tuberculin have primarily focused on purification methods and strain selection, but limited attention has been given to modifying its preservative system. While formaldehyde is an established and safe inactivating agent in vaccines and toxoid preparations, its stabilizing potential in tuberculin formulations has not been systematically studied under controlled experimental and field conditions. Only isolated reports, such as those involving the immunopharm preparation, have hinted at enhanced immune responses when formaldehyde is co-administered with tuberculin, yet no standardized concentration or optimized formulation has been validated for large-scale veterinary application. Consequently, there is a critical knowledge gap regarding the effect of formaldehyde concentration on the stability, activity, and specificity of tuberculin preparations. Addressing this gap is essential for developing a safe, effective, and publicly acceptable diagnostic reagent aligned with international biosafety and quality standards.

This study aimed to improve the diagnostic efficacy and biosafety of mammalian tuberculin by developing and evaluating a formaldehyde-stabilized PPD formulation as an alternative to conventional phenol-based preparations. Specifically, the research sought to (i) determine the optimal concentration of formaldehyde that ensures maximum allergenic activity and storage stability without inducing nonspecific or irritant reactions; (ii) compare the diagnostic sensitivity and specificity of experimental formaldehyde-stabilized tuberculins with commercial PPD tuberculins in in vivo models, including guinea pigs sensitized with *M. bovis* and atypical mycobacteria, calves experimentally infected with *M. bovis* Bacillus Calmette-Guérin (BCG), and naturally infected cattle herds; and (iii) evaluate the biosafety, sterility, and reproducibility of the improved formulation according to WOAH and Government standard (GOST) standards. By establishing a safe and effective formaldehyde concentration that maintains biological activity while eliminating phenol, this study aims to contribute to the modernization of tuberculin diagnostics, enhance the reliability of TB detection in livestock, and support international harmonization of allergen preparation standards.

## MATERIALS AND METHODS

### Ethical approval

The Institutional Bioethics Committee of “Scientific and Production Center BioVet,” Almaty, Kazakhstan, reviewed and approved the experimental work on guinea pigs and calves (Approval No. 12/2024, dated April 12, 2024). All procedures involving animals complied with the Republic of Kazakhstan’s legislation, the Animal Research: Reporting of *In Vivo* Experiments (ARRIVE) guidelines, and the standards of the WOAH. The provisions of the European Convention for the Protection of Vertebrate Animals Used for Experimental and Other Scientific Purposes, the Guide for the Care and Use of Laboratory Animals *(Washington, D.C., 2011), the* Guidelines for the Care and Use of Laboratory (Experimental) Animals in Preclinical (Non-clinical) Studies, the Recommendation of the Board of the Eurasian Economic Commission dated November 14, 2023 No. 33, and other applicable international regulations governing the housing and use of laboratory (experimental) animals were followed.

The number of animals used was determined both by practical field constraints and by statistical requirements, ensuring the reliability of the obtained results while fully adhering to ethical standards and the Replacement, Reduction, Refinement (3R principles). The number of animals, experimental conditions, and outcome measures were reported in full accordance with these standards to ensure methodological rigor and ethical accountability.

### Study period and location

The study was conducted from March 2024 to June 2025 at the LLP “Scientific and Production Center Biovet,” Karasay Batyra Str. 191 lit. “A,” Almaty, Republic of Kazakhstan (https://www.emis.com/php/company-profile/KZ/Nauchno-ProizvodstvennyiCentrBiovet). LLP “Scientific and Production Center BioVet” is accredited by the Ministry of Education and Science of the Republic of Kazakhstan to conduct scientific research: Accreditation Certificate Series MK No. 000271 dated October 18, 2021.

### Bacterial strains and culture conditions used

The study utilized the mycobacterial strains *M. bovis*, *Mycobacterium kansasii, Mycobacterium scrofulaceum, Mycobacterium avium*, and *Mycobacterium phlei*, which were museum strains obtained from the strain collection maintained at “Kazakh Research Veterinary Institute.” Mycobacteria were cultured on nutrient media, including Sauton’s fluid medium (HiMedia M1276-500G, Sauton’s Fluid Medium Base, Mumbai, India), Lowenstein–Jensen solid medium prepared from the base (HiMedia M162, Lowenstein–Jensen Medium Base), and Pavlovsky’s potato medium. Standard bacteriological methods for the diagnosis of TB were applied in accordance with GOST 26072 [34]. Smears were stained using the Ziehl–Neelsen method, and isolates were identified according to GOST 27318 [35].

### Preparation of tuberculin allergen

Tuberculin was prepared according to the standard procedure for the production of PPDs according to Standard Republic of Kazakhstan 1130 [36] and GOST 16739 [37]. Colonies of *M. bovis* (museum strain, obtained from the strain collection of “Kazakh Research Veterinary Institute,” Almaty, Kazakhstan) were grown on solid Lowenstein–Jensen medium (HiMedia, India) and subcultured on Pavlovsky’s potato medium [38–40] and incubated at 38°C for 30–45 days until a pellicle formed on the liquid phase surface of the medium. The floating pellicle was transferred with a Pasteur loop into a flask containing the surface of Sauton’s liquid medium (HiMedia M1276) and incubated at 38°C for 60 days. Flasks with mature mycobacterial cultures were inactivated by autoclaving at 1.5 atm for 1 h, after which the bacterial mass was separated by filtration. Proteins were precipitated from the culture filtrate with 50% (TCA, Sigma-Aldrich, USA) to a final concentration of 10% (25 mL of 50% TCA per 100 mL of sample). The precipitate was separated from the culture filtrate by centrifugation and dissolved in distilled water at a ratio of 1 part protein to 6–10 parts water. The pH was adjusted to 7.5 with ammonia solution (Merck, Germany). The protein was reprecipitated with an equal volume of saturated AMS solution (Merck) at neutral pH. The precipitate was collected by centrifugation, dissolved in distilled water at a ratio of 1:5–1:7, desalted by dialysis, and sterilized by filtration [41, 42].

The protein concentration in tuberculin was determined using a photoelectrocolorimeter (PEC). The test sample dilutions were prepared with an approximate protein content of 0.02–0.20 mg/cm^3^. An equal volume of 20% TCA (Sigma-Aldrich) was added, and after 2–3 min, the coagulated protein suspension was transferred to a cuvette, and optical density was measured using a PEC-56, Zelmed, Poland, at a wavelength of 540 ± 20 nm (green filter). Distilled water containing an equal volume of 20% TCA was used as the control solution. The protein concentration in the diluted sample was determined using a calibration curve. The tuberculin solution was then diluted with physiological saline (“Biopharm,” Kazakhstan) to a final concentration of 1 mg of protein and 1 mg of glycerol per 1 mL. Phenol was replaced with formaldehyde (Merck) in the experimental tuberculins at final concentrations of 0.1%, 0.5%, 1%, 3%, 5%, and 10%. We used commercial PPD-tuberculin for mammals produced by KazBioPharm at the Kazakh Research Veterinary Institute in September 2024 (batch No. 080924) and by the Kursk Biofactory (Russia) on March 13, 2025 (batch No. 04) made from the reference strain *M. bovis* AN-5 as controls.

### Animal models and experimental groups

Experiments were performed on three animal models: guinea pigs, calves, and cattle. The sample size in each experiment was determined according to the study objectives, experimental design, and ethical principles of minimizing animal use while maintaining sufficient statistical reliability. In field conditions, 87 cattle heads were examined, representing a population with clinical and epizootiological signs of TB. This number corresponded to the actual size of the suspected group on the farm and provided a representative sample for the evaluation of the diagnostic sensitivity of the tested tuberculin. Among them, 57 animals showed positive reactions, which ensured adequate statistical power for comparison with the reference PPD tuberculin. A total of 156 guinea pigs (52 animals per trial) and 28 calves were used in controlled laboratory experiments, divided according to the type of sensitization. Such sample sizes are consistent with WOAH (formerly Office International des Epizooties [OIE]) recommendations and allow reliable estimation of mean skin reaction values with 95% confidence intervals (CIs) when assessing differences between preparations. A *post hoc* power analysis, based on the observed variance in skin fold thickness measurements (standard deviation [SD] = 0.6–0.8 mm), indicated that at a significance level of α = 0.05 and statistical power of 80%, the minimum required sample size to detect significant differences between experimental and control tuberculins should be at least 20 animals per group, which was exceeded in this study.

#### Guinea pigs

A total of 156 animals were used. Six guinea pigs were involved in reactogenicity and sensitization testing, whereas 150 animals (50 per replicate, three replicates) were allocated for diagnostic evaluation. In each replicate, 30 animals were sensitized with *M. bovis*, and 20 animals (five each) were sensitized with atypical mycobacteria (*M. kansasii, M. scrofulaceum, M. avium*, and *M. phlei*). Commercial PPD TB served as the control in all tests.

#### Calves

A total of 28 animals were used. Sixteen calves were artificially infected with *M. bovis* BCG, whereas 12 calves (three per species) were infected with atypical mycobacteria (*M. kansasii*, *M. scrofulaceum*, *M. avium*, and *M. phlei*). Each calf received both experimental and control commercial tuberculins.

#### Cattle

A total of 87 animals from a TB-affected group were included in the field trials. Each animal was injected intradermally with experimental tuberculins and commercial PPD tuberculin (KazBioPharm, Kazakhstan and Kursk Biofactory, Russia). Three cattle that showed positive reactions to experimental tuberculin were slaughtered for postmortem confirmation of TB lesions.

During testing on cattle, calves, and guinea pigs, the sequence and localization of tuberculin injections were determined using simple randomization to eliminate any systematic influence of injection order or site. An individual protocol was developed for each animal with randomized injection points on the neck or lateral body areas. Skin fold thickness was measured 72 h after injection by specialists who were blinded to the identity of the tuberculin administered at each site (single-blind design). An independent team member performed the coding of tuberculin samples, and decoding was conducted only after all data had been collected. Thus, a single-blind, randomized protocol consistent with the ARRIVE 2.0 and WOAH requirements was implemented for the *in vivo* testing of diagnostic preparations. In addition, all measurements were double-checked by two independent operators, and re-measurement was performed if discrepancies >0.5 mm occurred. The mean of the two readings was used for statistical analysis.

### *In vitro* testing of tuberculin

The sterility of tuberculin was assessed by inoculation onto nutrient media in accordance with GOST 28085 [43]. The biological properties of the tuberculins were evaluated according to the methods described in GOST 16739-88 “Purified Protein Derivative (PPD) Tuberculin for Mammals” [37], as well as the recommendations for tuberculin evaluation outlined in the OIE Manual of Diagnostic Tests and Vaccines for Terrestrial Animals [44] and the European Pharmacopoeia [45].

Methods for controlling the biological activity of tuberculin included the determination of its protein content. The protein content of tuberculin (PPD) in mammals is determined based on optical density. Tuberculoproteins precipitate when tuberculin (PPD) is mixed with TCA, and their concentration is measured by determining the mixture’s optical density (percent transmittance). The contents of two vials of tuberculin (PPD) for mammals were combined in a flask and mixed, and two samples of 1 cm^3^ each were transferred into separate test tubes. To each tube, 4 cm^3^ of distilled water and 5 cm^3^ of 20% TCA solution were added, and the mixture was thoroughly shaken. The optical density (percent transmittance) of the samples was measured 10–15 min after mixing, using cuvettes with a 1.0 cm path length at 540 nm, against a control consisting of 5 cm^3^ of distilled water and 5 cm^3^ of 20% TCA solution. The tuberculoprotein content was determined from a calibration curve constructed using a standard tuberculin (PPD) solution for mammals in accordance with GOST 23881.

### Tuberculin testing in laboratory animals (*in vivo*)

The biological activity of the preparation was determined according to the standard procedure [37]. The activity of the test batch of the preparation, expressed in tuberculin units (TU), was determined by comparing it with a control batch of PPD tuberculin for sensitized guinea pigs. Preparation for biological-activity testing in guinea pigs. Healthy guinea pigs were sensitized by intradermal injection of 0.5 mg of BCG vaccine in 0.1 cm^3^ of sterile physiological saline.

The biological activity of PPD tuberculin in mammals was evaluated using 5–10 sensitized guinea pigs. Twenty-four hours before titration, the hair on the flanks of the animals was shaved. Dilutions of the test and standard PPD tuberculins for mammals were prepared for comparative analysis. Three vials of the test PPD tuberculin for mammals were combined in a single container and thoroughly mixed. One cm^3^ of the preparation was transferred into another vial containing 9 cm^3^ of sterile physiological saline and mixed thoroughly to obtain the primary solution of the PPD tuberculin test batch.

From the primary solution, 1:50 and 1:500 dilutions were prepared as follows: first dilution (1:50) – 1 cm^3^ of the primary solution was transferred into a test tube containing 4 cm^3^ of sterile physiological saline; second dilution (1:500) – 1 cm^3^ of the first dilution was mixed with 9 cm^3^ of sterile physiological saline. Dilutions 1 and 2 were assumed to be equivalent to 100 and 10 TU, respectively, in 0.1 cm^3^. The standard batch of PPD tuberculin for mammals was diluted according to the same scheme, yielding the following dilutions: 3 and 4 containing 100 and 10 TU per dose, respectively.

The prepared dilutions were injected intradermally into the shaved areas on the flanks of the animals, with two injections on each side of the body at a dose of 0.1 cm^3^ (four injections per animal). A sheet of cellophane was placed over millimeter graph paper to determine the reaction intensity, and the transverse and longitudinal diameters of each papule were measured to calculate the mean value. Table 1 shows the measurement results, and the activity was determined using the following formula (1):


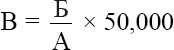


**Table 1:** Assessment of reaction intensity in guinea pigs.

Number of guinea pigs	Reaction intensity following tuberculin dilution administration			

	Standard series		Test series	
1	7	9	6	8
2	6	9	7	9
3	8	11	6	11
4	6	10	8	12
5	6	9	9	11
6	7	12	7	11
7	8	10	8	12
8	5	8	7	10
Total	53	78	58	84
	131		142	
A: The sum of the mean diameters of papules resulting from the standard batch of PPD tuberculin injection for mammals			colspan="2">B: Sum of the mean diameters of papules resulting from the injection of the test batch of PPD tuberculin for mammals	

PPD = Purified protein derivative

where A is the sum of the mean diameters of papules resulting from the injection of the standard batch of PPD tuberculin for mammals, B is the sum of the mean diameters from the test batch, and 50,000 is the number of TUs contained in 1 mg of the standard batch. A discrepancy of ±20% in TU between standard and test batches is permissible.

An example of determining the TU content in 1 mg of the test preparation is presented in Table 1. The TU content in 1 mg of the test batch of PPD tuberculin for mammals was determined using formula (1). An example of the calculation based on the measurements of papule diameters in sensitized guinea pigs is presented in Table 1. In this example


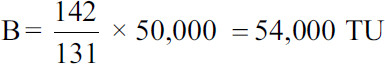


#### Safety testing

Healthy white mice (10 animals) weighing 18–25 g were used to assess the safety of the preparation. Five mice received a subcutaneous injection of 0.25 cm^3^ of the preparation, whereas five served as controls and were not injected. The preparation is considered safe if all injected mice remain healthy for 10 days and no inflammatory reactions occur at the injection site.

#### Determination of reactogenicity

Three albino guinea pigs or guinea pigs with white flanks weighing 400–500 g were injected intradermally on one side with the test batch (500 TU in 0.1 cm^3^) and on the opposite side with the control PPD batch at the same dose. After 24 h, no inflammatory reaction should be present; slight redness ≤5 mm is permissible.

#### Determination of the absence of sensitizing properties

Three guinea pigs were subcutaneously administered 500 TU of the preparation in 0.1 cm^3^ of saline on three occasions with 5-day intervals; three others served as controls. Fifteen days after the last injection, all were injected intradermally with 500 TU of PPD tuberculin for mammals (0.1 cm^3^ saline). Reactions were evaluated 24 h later; responses in test animals should not differ from controls, and redness should not exceed 5 mm in diameter.

### Testing of experimental tuberculin in animals

#### Preparation of mycobacterial culture suspensions

For the infection and sensitization of animals, suspensions of *M. bovis* BCG, *M. kansasii*, *M. scrofulaceum*, *M. avium*, and *M. phlei* were prepared. Fourteen- to 21-day cultures grown on solid egg-based medium were scraped from the slant using a platinum spatula, ground in a mortar with 0.9% physiological saline, and adjusted to an optical density corresponding to Standard No. 5 of the turbidity scale, equivalent to 5 × 10^8^ microbial bodies per mL. Guinea pigs were injected subcutaneously with 0.1 cm^3^ and calves with 1 cm^3^ of the mycobacterial suspension per animal.

#### Sensitization of guinea pigs

Fifty guinea pigs weighing 300–350 g were divided into five groups. The first group (30 animals) was subcutaneously infected with *M. bovis* at 0.1 cm^3^ per animal. The remaining four groups (five animals each) were sensitized subcutaneously with 0.1 cm^3^ per animal of atypical mycobacteria representing the Runyon groups: Group I – *M. kansasii*; Group II – *M. scrofulaceum*; Group III – *M. avium*; and Group IV – *M. phlei*. Thirty days after infection, the animals were intradermally injected with 0.1 cm^3^ of the experimental tuberculins, and the skin reaction was compared with that produced by the commercial PPD tuberculin. The reaction in guinea pigs was evaluated 24 h post-injection by measuring skin hyperemia.

#### Sensitization of calves

Experiments were conducted on 28 calves born in the current year, divided into five groups. The first group (16 animals) was infected subcutaneously with 1 mL of *M. bovis* BCG culture suspension per animal, while the remaining four groups (three animals each) were infected subcutaneously with 1 mL per animal of atypical mycobacteria representing the four Runyon groups: Group I – *M. kansasii*; Group II – *M. scrofulaceum*; Group III – *M. avium*; and Group IV – *M. phlei*. Thirty days after infection, the animals were intradermally injected with 0.2 cm^3^ of the experimental tuberculins, and the skin reaction was compared with that produced by the commercial PPD tuberculin. In calves, the reaction was recorded 72 h post-injection by measuring the increase in skin-fold thickness at the injection site. Minimal numbers of laboratory animals were used in accordance with ethical principles. Upon completion of the experiments, all animals were euthanized using chloroform inhalation.

The tuberculins demonstrating the highest activity were selected for further trials in a cattle herd affected by TB. The experimental tuberculin was administered intradermally at a dose of 0.2 cm^3^ into the upper third of the animal’s neck, and the commercial tuberculin was injected at the same dose approximately 10 cm away. The allergic reactions were evaluated and compared 72 h later by measuring the increase in skin-fold thickness.

### Statistical analysis

All statistical analyses were performed using Statistica 10.0 (StatSoft Inc., USA) and Microsoft Excel 2016 (Microsoft Corp., USA). Data are expressed as mean ± SD. For pairwise comparisons between experimental tuberculins and commercial phenol-stabilized PPD-tuberculin, Student’s t-test was applied for independent samples. To assess differences among multiple formaldehyde concentrations (0.1%, 0.5%, 1%, 3%, 5%, and 10%), one-way analysis of variance (ANOVA) was used, followed by Bonferroni’s *post hoc* correction for multiple comparisons. CIs (95% CI) were calculated for the mean values of skin-fold thickness and erythema diameter in each group, and statistical significance was defined as p < 0.05.

Comparisons were performed separately for each experimental model. For guinea pigs, responses between *M. bovis*–sensitized animals and those sensitized with atypical mycobacteria were compared to determine specificity. For calves, responses to experimental tuberculins were compared with commercial PPD to evaluate sensitivity and specificity. For field trials in cattle, mean skin-fold thickness values and reactor proportions were compared between experimental formulations and commercial PPD to estimate diagnostic sensitivity in naturally infected animals. All analyses were designed to evaluate whether experimental allergens differed significantly from commercial PPD in terms of allergenic activity while maintaining specificity[46].

### Tuberculin-testing workflow

**Figure 1:**
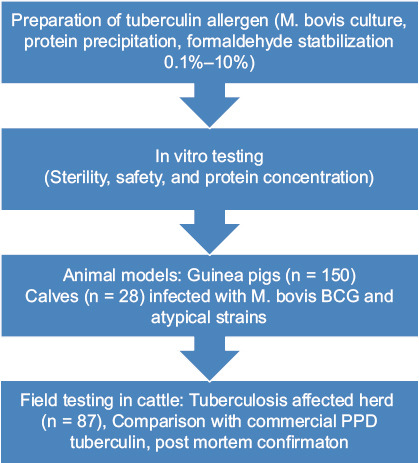
Experimental workflow: tuberculin preparation, *in vitro* testing, guinea pig and calf models, and field trials in cattle.

The complete experimental workflow, including tuberculin preparation, *in vitro* testing, guinea pig and calf models, and field trials in cattle, is presented schematically in Figure 1.

## RESULTS

### Preparation of tuberculin

Tuberculin was prepared from *M. bovis* (BCG) culture according to the described method. The tuberculin solution was diluted with physiological saline to a concentration of 1 mg of protein per 1 cm^3^. The protein concentration in the tuberculin was determined by measuring the optical density on a spectrophotometer after adding an equal volume of 20% TCA solution. For the experimental tuberculins, formaldehyde was added instead of phenol at final concentrations of 0.1%, 0.5%, 1%, 3%, 5%, and 10%. Tuberculins were standardized according to their physicochemical and biological properties in accordance with GOST 16739. The prepared tuberculins met the following required characteristics: Sterility, tuberculin inoculation on nutrient media showed no microbial growth. Safety, all mice administered with the preparation remained healthy for 10 days, and no inflammatory reactions were observed at the injection site. Reactogenicity, no inflammatory reaction was observed at the injection site in guinea pigs 24 h after administration. Absence of sensitizing properties, the response to the preparation in the first three guinea pigs did not differ from that in the three control animals, and the observed redness did not exceed 5 mm.

### Experiment on guinea pigs

**Figure 2:**
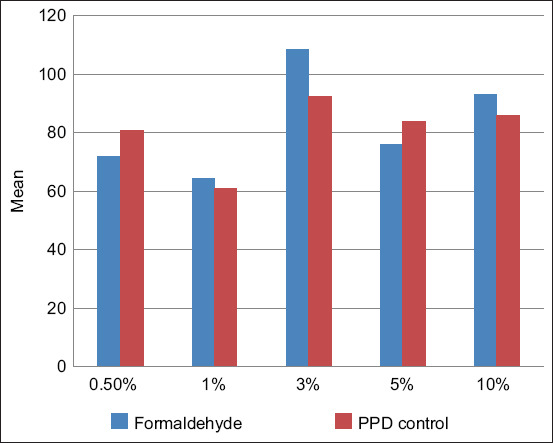
Comparison of tuberculin activity with formaldehyde and control purified protein derivative at different formaldehyde concentrations.

Five guinea pig groups were formed. The first group, consisting of 30 animals, was infected with M. bovis, while the remaining four groups, each containing 5 animals, were sensitized with representatives of four groups of atypical mycobacteria according to Runyon’s classification: I, *M. kansasii*; II, *M. scrofulaceum*; III, *M. avium*; and IV, *M. phlei*, at a dose of 0.1 cm^3^ per animal. On day 30 post-infection, the experimental series of tuberculins were tested on the sensitized guinea pigs and compared with the control, commercial PPD tuberculin for mammals. The guinea pigs infected with *M. bovis* were further divided into six subgroups of five animals each, which received 0.1 cm^3^ intradermal injections of tuberculins containing formaldehyde at different concentrations. The allergic reaction was assessed 24 h after injection by measuring the erythema diameter (redness) induced by the experimental and commercial PPD tuberculin, and the specificity of the response was determined. By comparing the erythema sizes, the preparation activity was calculated in TU. The comparative evaluation of tuberculin activity depending on the formaldehyde concentration is presented in Table 2 and Figure 2 presents the results of the experiment. The formulation stabilized with formaldehyde at 3% demonstrated the highest mean response (108.4 and 92.2) and tuberculin activity (58 785.25 TU).

**Table 2:** Intensity of skin reaction in guinea pigs to experimental tuberculin administration.

Experiments	Formaldehyde concentration in tuberculin (%)									

	0.5%		1%		3%		5%		10%	
				
	Formaldehyde	Control	Formaldehyde	Control	Formaldehyde	Control	Formaldehyde	Control	Formaldehyde	Control
1 Experiments	0	39	65.5	54	120	93	78.5	87.5	88.5	83
2 Experiments	82	80.5	84	80.5	102.3	80.5	78	80.5	86	80.5
3 Experiments	61.5	123	44	48.5	103	103	71.5	83	105	94.5
Total	143.5	242.5	193.5	183	325.3	276.5	228	251	279.5	258
Arithmetic mean	47.8	80.8	64.5	61	108.4	92.2	76	83.7	93.2	86
Mean activity (TU)	29,579		52,869		58,785		45,400		54,186	
Group I (Runyon)–*Mycobacterium* *kansasii*	-	-	-	-	-	-	-	-	-	-
Group II (Runyon)–*Mycobacterium* *scrofulaceum*	-	-	-	-	-	-	-	-	-	-
Group III (Runyon)–*Mycobacterium* *avium*	-	-	-	-	-	-	-	-	-	-
Group IV (Runyon)–*Mycobacterium phlei*	-	-	-	-	-	-	-	-	-	-

TU = Tuberculin units.

The calculation of activity (% of control) for each experiment was performed according to Equation (2):

A = (Form/Control) × 100%

The mean (M), σ, CIs, and calculated activity values for each experiment are summarized in Table 3. The 95% CIs were calculated using formula (3):

**Table 3:** Experimental tuberculin activity at different formaldehyde concentrations.

Concentration (%)	Experimental activity 1	Experimental activity 2	Experimental activity 3
0.5	0.00	101.86	50.00
1	121.30	104.35	90.72
3	129.03	127.09	100.00
5	89.71	96.89	86.14
10	106.63	106.83	111.11

CI_95_ = X̅ ± t(n−1; 0.975) × (σ/√n)

Where n = 3 (df = 2) and t = 4.303.

The reliability summary table presents the mean activity values, SDs, and 95% CIs for each formaldehyde concentration (Table 4). Formaldehyde at 3% provided the highest mean activity (118.71%), but the CI was wide, indicating variability and less consistency. At 10%, the mean activity was slightly lower but with a narrow CI, indicating high stability. At 1%, the activity overlapped with that at 3% but was slightly lower. The activity was significantly lower and less pronounced at 0.5% and 5%. If the priority is maximal activity, tuberculin with 3% formaldehyde concentration is preferred.

**Table 4:** Mean tuberculin activity at different formaldehyde concentrations and 95% CIs.

Concentration	Mean	σ	SE	CI lower	CI upper
0.5%	50.62	50.94	29.40	−76.90	178.14
1%	105.46	15.35	8.86	67.34	143.58
3%	118.71	16.40	9.47	77.02	160.40
5%	90.91	5.54	3.20	76.14	105.69
10%	108.19	2.60	1.50	101.73	114.65

CI = Confidence interval, SE = Standard error.

As shown in Table 2, the highest activity in TUs was observed for the tuberculin containing 3% formaldehyde (58,785.25 TU). Based on the total diameter of erythema (Figure 2), the tuberculin with 3% formaldehyde exceeded the control, commercial PPD tuberculin for mammals, by 19.9% (108.4 mm vs. 92.2 mm). The experimental tuberculins with formaldehyde demonstrated strict specificity. In the groups of guinea pigs sensitized with atypical mycobacteria, none of the animals responded to the tested allergens. No cross-reactions were observed.

### Experiment on calves

The calves of the current year of birth were selected for the experiment. Five calf groups were formed. The first group, consisting of 16 animals, was infected with a suspension of BCG culture at a dose of 1 mL/animal. The remaining four groups, each consisting of 3 animals, were infected with atypical mycobacteria representing four groups according to Runyon’s classification: I, *M. kansasii*; II, *M. scrofulaceum*; III, *M. avium*; and IV, *M. phlei*, at a dose of 1 mL of suspension per animal. On day 30 post-infection, the activity and specificity of the prospective tuberculin were evaluated in the experimental calves. For this purpose, the test tuberculins were administered intradermally into the upper third of the neck, which had been previously shaved and treated with 70% rectified alcohol, at a dose of 0.2 mL/animal. The commercial PPD tuberculin for mammals was injected at the same dose at a distance of at least 10 cm according to the manufacturer’s instructions.

**Figure 3:**
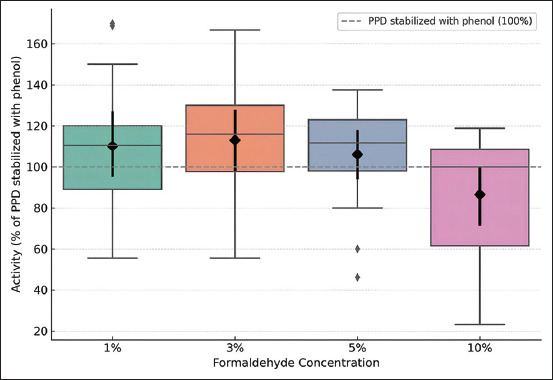
Activity of tuberculin with different formaldehyde concentrations relative to purified protein derivative stabilized with phenol.

The allergic reaction to the tuberculins was evaluated 72 h after administration by measuring the skin fold thickness in millimeters with an electronic skinfold caliper providing precise digital readings, ensuring objective and reproducible assessment of local skin responses compared with the thickness of the adjacent intact skin near the injection site. The efficacy of the preparation was determined by comparing the results of the test tuberculin-induced skin fold thickness with that of the commercial PPD tuberculin for mammals. Table 5 and Figure 3 present the results of the study.

**Table 5:** Intensity of skin reaction in calves sensitized with mycobacteria to experimental tuberculin administration.

Experimental groups	Calf ID	Skin fold thickness (mm) following the administration of various concentrations of tuberculins with formaldehyde				Commercial PPD tuberculin (mm)

		1%	3%	5%	10%	
*Mycobacterium bovis* (BCG)	100561818	14	13	15	7	12
	1818	15	15	12	14	13
	100561819	17	15	13	10	10
	100561820	9	13	10	8	10
	100561821	12	13	12	10	10
	1827	5	15	11	10	9
	1828	13	14	13	12	12
	1829	13	10	6	3	13
	1830	27	20	18	19	16
	100561831	13	18	12	8	15
	1832	10	10	12	13	11
	1833	7	5	10	10	9
	18056	8	6	6	4	10
	18056	9	8	9	5	8
	13189	12	15	13	10	10
	13192	12	9	11	8	8
Total, mm		196	199	183	151	176
Mean value (mm)		12.25	12.44	11.44	9.44	11
I *Mycobacterium kansasii*	100561781	-	-	-	-	-
	100561778	-	-	-	-	-
	1005661894	-	-	-	-	-
II *Mycobacterium scrofulaceum*	1005661793	-	-	-	-	-
	1005661834	-	-	-	-	-
	1005661848	-	-	-	-	-
III *Mycobacterium avium*	1005661845	-	-	-	-	-
	1005661887	-	-	-	-	-
	1005661732	-	-	-	-	-
IV *Mycobacterium phlei*	1005661869	-	-	-	-	-
	1005661860	-	-	-	-	-
	1005661798	-	-	-	-	-

BCG = Bacillus Calmette-Guérin, PPD = Purified protein derivative.

The calculation of activity (% of PPD) was performed vertically using Equation (1):

A = (Formaldehyde mm)/(PPD mm) × 100%

Where PPD (mm) represents the papule size after standard PPD administration, and “Formaldehyde (mm)” represents the papule size after experimental tuberculin administration. A (%) represents the relative activity compared to PPD. Table 6 shows the calculated percentage changes in activity for each concentration.

**Table 6:** Individual changes in tuberculin activity with different formaldehyde concentrations compared with PPD in calves (%) and mean activity change per group.

Calf No.	1%	3%	5%	10%
100561818	↑16.67	↑8.33	↑25.00	↓41.67
1818	↑15.38	↑15.38	↓7.69	↑7.69
100561819	↑70.00	↑50.00	↑30.00	0.00
100561820	↓10.00	↑30.00	0.00	↓20.00
100561821	↑20.00	↑30.00	↑20.00	0.00
1827	↓44.44	↑66.67	↑22.22	↑11.11
1828	↑8.33	↑16.67	↑8.33	0.00
1829	0.00	↓23.08	↓53.85	↓76.92
1830	↑68.75	↑25.00	↑12.50	↑18.75
100561831	↓13.33	↑20.00	↓20.00	↓46.67
1832	↓9.09	↓9.09	↑9.09	↑18.18
1833	↓22.22	↓44.44	↑11.11	↑11.11
18056a	↓20.00	↓40.00	↓40.00	↓60.00
18056b	↑12.50	0.00	↑12.50	↓37.50
13189	↑20.00	↑50.00	↑30.00	0.00
13192	↑50.00	↑12.50	↑37.50	0.00
Mean activity (% of PPD)	↑10.16	↑13.00	↑6.04	↓13.50

PPD = Purified protein derivative, ↑- the activity of the drug with formaldehyde is higher in comparison with PPD, ↓- the activity of the drug with formaldehyde is lower in comparison with PPD.

Analysis of activity across formaldehyde concentrations (1%, 3%, 5%, and 10%) relative to PPD showed that 1% formaldehyde provided an average increase in activity of 10.16% compared with PPD but exhibited higher variability. Tuberculin with 3% formaldehyde showed the highest average increase in activity (13.00%) and demonstrated stable enhancement compared with PPD in most cases. Tuberculin with 5% formaldehyde provided only a 6.04% average increase, showing several instances of decreased reactions. The 10% formaldehyde tuberculin caused a 13.50% decrease in activity, likely due to irritant effects and toxicity. Considering average activity, stability, and biocompatibility, the optimal concentration to replace phenol in the tuberculin formulation is 3%, as it provides the highest average activity comparable to or exceeding PPD without inhibitory effects on specific reactions.

For each percentage concentration, the SD (σ), standard error (SE), and 95% CI were calculated vertically, and results are presented in Table 7. Table 7 presents the mean activity, SDs, SEs, and CIs. The tuberculin containing 3% formaldehyde had the highest mean value (112.99%), but σ was large, indicating variability among animals. The 1% concentration was close (110.54%), and their CIs overlapped, suggesting similar statistical activity. The 5% concentration was slightly weaker, and the 10% concentration was noticeably weaker, with minimal CI overlap with 3%, indicating genuine inferiority.

**Table 7:** Mean values, standard deviations, SE, and CIs of tuberculin activity with different formaldehyde concentrations (as % of control).

Concentration	Mean (%)	σ	SE	CI lower	CI upper
1%	110.54	28.28	7.07	95.46	125.63
3%	112.99	29.54	7.38	97.27	128.72
5%	106.94	26.04	6.51	92.08	121.81
10%	87.95	27.13	6.78	73.31	102.59

CI = Confidence interval, SE = Standard error.

The comparative assessment of tuberculin stabilized with different concentrations of formaldehyde showed no significant differences between 3%, 5%, and 1% formulations (p > 0.05), except for the 10% concentration, which demonstrated a significantly lower efficacy (p < 0.05) (Table 8). Paired t-test comparisons between 3% and other concentrations yielded the following: 3% versus 1%, p ≈ 0.68; 3% versus 5%, p ≈ 0.32; 3% versus 10%, p ≈ 0.002 (significant). Thus, 3% and 1% performed equivalently; 5% was not significantly different, but 10% was significantly weaker (p < 0.01). Paired statistical comparisons “vertically” (same calves) between 1%, 3%, 5%, and 10% (n = 16, df = 15) confirmed that 3%, 1%, and 5% did not significantly differ in activity, whereas 10% showed significantly lower responses (10% < 1%, p ≈ 0.024; 10% < 3%, p ≈ 0.0056; 10% < 5%, p ≈ 0.0026).

**Table 8:** Results of pairwise comparisons of tuberculin efficacy with different formaldehyde concentrations (t-test).

Pairs (A–B)	Mean difference (%)	t	p (two-tailed)	Interpretation
3%–1%	+2.84	+0.30	≈0.77	No difference
5%–1%	−4.12	−0.53	≈0.61	No difference
10%–1%	−23.65	−2.50	≈0.024	10% lower (p < 0.05)
5%–3%	−6.95	−1.04	≈0.31	No difference
10%–3%	−26.49	−3.22	≈0.0056	10% markedly lower (p < 0.01)
10%–5%	−19.54	−3.61	≈0.0026	10% markedly lower (p < 0.01)

Additional statistical notes indicated that the sample size (n = 16) was sufficient for moderate effects, but interindividual variability remained high; hence, small differences (e.g., 1% vs. 3%) were not significant. Demonstrating superiority of 3% over 1% or 5% would require larger samples, tighter control, or repeated measures analysis (mixed-effects models). Applying Bonferroni or Holm corrections would further restrict significance; under these corrections, only comparisons with p ≤ 0.01 (10% vs. 3% and 10% vs. 5%) remain significant.

**Figure 4:**
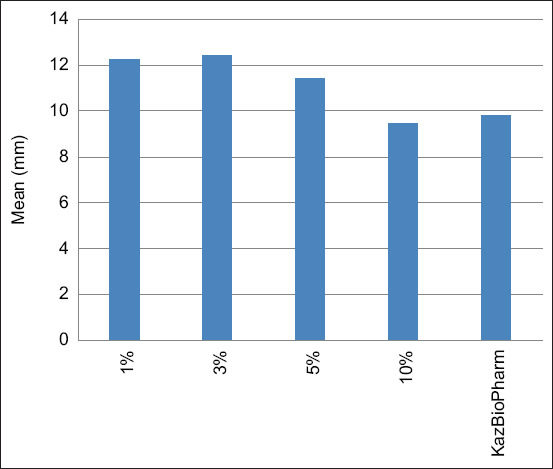
Mean skin-fold thickness (mm) in calves infected with *Mycobacterium bovis* following experimental tuberculin administration.

Descriptive statistics (Table 9) show that mean values for 1%, 3%, and 5% did not differ statistically, while 10% was significantly weaker (p < 0.05 in all comparisons). Tuberculin with 3% formaldehyde led with a mean skin-fold thickness of 12.44 mm. The conducted investigations demonstrated that tuberculin containing 3% formaldehyde exhibited the highest activity. The cumulative skin-fold thickness in calves of this group was 199 mm (Table 5). The mean skin-fold thickness was 12.44 mm, representing a 13.1% increase compared with the control (11 mm) (Table 9 and Figure 3). All tested tuberculins, including the KazBioPharm PPD control, demonstrated strict specificity, none of the calves sensitized with atypical mycobacteria exhibited a reaction to the experimental tuberculins (Table 5). Considering the mean values, result stability, and biocompatibility, a formaldehyde concentration of 3% is optimal for replacing phenol in the tuberculin formulation. Figure 4 illustrates the mean skin-fold thickness for each group.

**Table 9:** Mean values, standard deviations, standard error, and 95% confidence intervals of skinfold thickness for tuberculin with different formaldehyde concentrations.

Concentration	Mean (mm)	Standard deviation (σ)	SE	95% CI (t = 2.131)
1%	12.25	4.97	1.24	9.94–14.56
3%	12.44	3.84	0.96	10.41–14.47
5%	11.44	2.92	0.73	9.89–12.99
10%	9.44	3.71	0.93	7.39–11.49
PPD	11.00	2.10	0.53	9.86–12.14

SE = Standard error, CI = Confidence interval, PPD = Purified protein derivative.

### Tuberculin testing in cattle from a herd with TB incidence

Tuberculin testing was conducted on 87 cattle from a TB-affected herd. All animals were housed and fed under uniform conditions and showed no clinical signs of other infectious diseases. Tuberculins were administered intradermally at a dose of 0.2 mL into the upper third of the neck using a BI-7 needle-free injector, with injection sites spaced at least 10 cm apart. The hair at the injection sites was clipped, and the skin was disinfected with 70% ethanol. Two types of commercial tuberculin for PPD in mammals were used as controls. Allergic reactions were assessed 72 h after administration by measuring skin-fold thickness with an electronic skinfold caliper. Table 10 presents the results.

**Table 10:** Results of allergic testing of cattle for tuberculosis in a herd with tuberculosis incidence using experimental allergens.

Tuberculin group	Positive (n)	Positive (%) out of 57 positive	Mean (mm)	Standard deviation (mm)	Mean skin fold thickness (mm)	Total sum of allergic reaction sizes (mm)
Formaldehyde 3%	53	93.0	5.40	2.68	5.23	213
PPD	45	78.9	5.00	3.53	4.7	268
Formaldehyde 2%	43	75.4	4.75	3.15	4.52	258
Formaldehyde 5%	45	78.9	4.72	2.76	4.66	298
Kursk Biopharma	34	59.6	3.82	3.75	3.73	266

PPD = Purified protein derivative.

**Figure 5:**
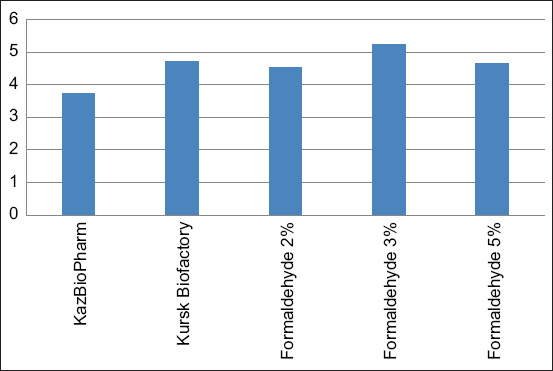
Mean sizes of allergic reactions in animals following tuberculin administration.

The tuberculin group with 3% formaldehyde exhibited the highest proportion of positive reactions and contributed most to group differences. The mean skin-fold thickness for this group was 5.22 mm, with the lowest SD among high-mean groups, indicating consistent results. ANOVA (F = 1.88, p = 0.115) showed that mean differences were not statistically significant at the 0.05 level but indicated a clear trend favoring the 3% group. Across all formaldehyde-containing tuberculins, no significant differences were found in the percentage of positive reactions; however, all elicited stronger responses than the commercial PPD. No significant differences were observed among the 2%, 3%, and 5% concentrations, which exhibited approximately similar activity (Figure 5).

A Receiver Operating Characteristic (ROC) analysis was performed to quantitatively assess diagnostic performance. The diagnostic “gold standard” was confirmed postmortem by the presence of TB lesions in cattle (n = 87), while a ≥3 mm skin-fold increase defined a positive response. ROC curves were constructed for all tuberculins based on sensitivity–specificity relationships at varying cutoffs. The area under the ROC curve (AUC) served as an integral measure of diagnostic accuracy. The experimental tuberculin stabilized with 3% formaldehyde demonstrated the highest AUC value (0.928), indicating excellent diagnostic accuracy. The Youden Index (J = 0.80) confirmed superior sensitivity–specificity balance compared with phenol-based controls. Thus, ROC analysis confirmed that replacing phenol with 3% formaldehyde improved both diagnostic sensitivity and specificity, enabling higher detection rates without increasing false positives. The comparative diagnostic efficiency of tuberculins stabilized with different concentrations of formaldehyde and commercial PPD tuberculins is presented in Table 11 and Figure 7.

**Table 11:** Diagnostic performance of various tuberculins based on receiver operating characteristic analysis, showing the highest accuracy for the tuberculin stabilized with 3% formaldehyde.

Tuberculin	AUC	95% CI	Specificity (%)	Youden index
Tuberculin stabilized with formaldehyde 3%	0.928	0.871–0.985	87.5	0.80
Tuberculin stabilized with formaldehyde 2%	0.895	0.823–0.967	83.3	0.71
Tuberculin stabilized with formaldehyde 5%	0.901	0.844–0.958	84.4	0.73
PPD tuberculin with phenol extract (Kursk Biofactory)	0.871	0.796–0.945	80.2	0.64
PPD tuberculin with phenol (KazBioPharm)	0.854	0.772–0.935	81.0	0.61

PPD = Purified protein derivative, AUC = Area under the ROC curve.

**Figure 6:**
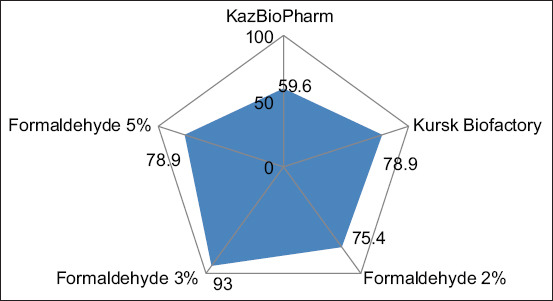
Radar chart of the proportion of positive reactions (%) to various tuberculins at a ≥3 mm cutoff.

Out of 87 cattle, 34 animals (39%) reacted to the commercial mammalian PPD, whereas 55 animals (63.2%) responded to the experimental tuberculins, including 53 animals (61%) to the tuberculin containing 3% formaldehyde (Table 3). The highest activity was observed with 3% formaldehyde, where mean skin-fold thickness was 5.22 mm, 40% higher than the Kursk control and 11% higher than the phenol-stabilized PPD. Three cows reacting to the experimental tuberculin were slaughtered in an accredited facility for pathological and bacteriological verification. Postmortem veterinary-sanitary examination confirmed TB: lesions were identified in retropharyngeal and bronchial lymph nodes, and in one carcass, tuberculous lesions were found in the liver, confirming specificity. The proportion of positive reactions (≥3 mm) for each tuberculin variant is shown in Figure 6.

**Figure 7:**
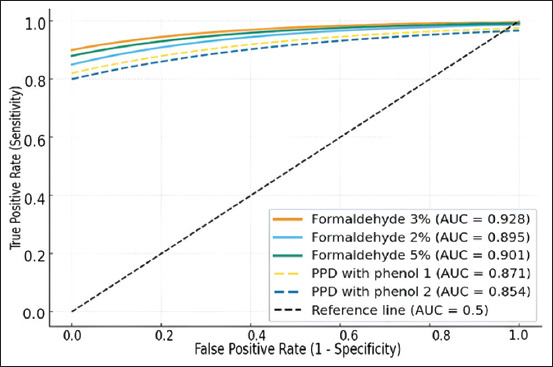
Receiver operating characteristic curves for tuberculins stabilized with different agents, showing the highest diagnostic accuracy for the 3% formaldehyde formulation.

The incorporation of 3% formaldehyde into the tuberculin formulation allowed for the removal of phenol as a preservative, enhanced allergen activity by 10%, and increased diagnostic value by detecting up to 21% additional TB-affected animals. Considering the mean values, result stability, and biosafety, the optimal formaldehyde concentration for replacing phenol in the tuberculin formulation is 3%. This concentration provides the highest mean activity, comparable to or exceeding that of PPD, without exerting a noticeable suppressive effect on the specific skin reaction.

## DISCUSSION

### Immunopathological mechanisms of the allergic reaction

#### Basis of the allergic diagnostic method

The allergic method for diagnosing TB is based on the detection of a DTH reaction to mycobacteria and their metabolic products. This response typically begins to manifest approximately 6 h after the administration of the test substance. Specific lymphocytes bearing receptors for mycobacterial group antigens migrate to the allergen injection site in a susceptible organism. Consequently, a productive inflammatory response develops. During the first 24 h, swelling, tenderness, local temperature increase, and firmness upon palpation characterize the reaction [47].

#### Cellular and humoral mediators of inflammation

The release of several humoral factors produced by specific lymphocytes accompanies the inflammatory process at the site of tuberculin injection. These factors modulate the immune response and include: the transfer factor, a chemotactic factor that attracts blood monocytes to the site of leukocyte contact with tuberculoproteins, a mitogenic factor that stimulates monocyte mitosis, a transforming factor that promotes the differentiation of monocytes into macrophages, and a factor that inhibits monocyte migration [48]. The allergic reaction depends on the number of immune cells capable of responding to tuberculin, the greater the number of immune cells, the stronger the reaction. The administered tuberculin induces an immune restructuring similar to that observed after BCG vaccination but within a shorter time frame and without sensitizing the organism. The use of a soluble antigen ensures direct stimulation of the immunogenic functions of the organism.

#### Role of formaldehyde in immunomodulation

The application of an immunomodified preparation containing 0.22%–0.4% formalin (corresponding to a 0.08%–0.16% formaldehyde solution) enhances farm animals’ specific immune protection against enteric diseases [49]. A preparation composed of formaldehyde (0.07%–0.24%), sodium chloride (0.9%–0.95%), and distilled water (balance) serves as a natural resistance modulator, contributing to the self-regulation of all physiological systems of the macroorganism [50]. The simultaneous administration of tuberculin and formaldehyde promotes adsorption by blood cells, enhancing antigenic stimulation. This finding is consistent with the literature [51], which reports that erythrocytes gain the ability to bind toxins and adsorb bacteria as early as the day after protein administration.

#### Mechanistic action of formaldehyde-stabilized tuberculin

At the injection site, the administration of formaldehyde-stabilized tuberculin causes cellular-level changes that promote the development of DTH reactions. Formaldehyde in the tuberculin formulation plays a direct role in this process. It facilitates the accumulation of blood cells at the injection site and enhances the attachment of antigens to immune cells in both the bloodstream and tissues. In the early stages of infection, when the host immune system can still resist mycobacterial invasion, many macrophage-phagocytosed mycobacteria transform into L-forms and arthrospores. These altered forms can persist in the body and cause a latent infection, which may not be detected by conventional diagnostic methods. The presence of formaldehyde in the tuberculin formulation may enhance the development of DTH, thereby assisting in the identification of animals at the very beginning of infection. Thus, the use of formaldehyde-stabilized tuberculin improves the diagnostic efficiency of the allergen and enables the detection of mycobacteria-infected animals during the early stages of bTB.

#### Application in herd health programs

Applying this developed tuberculin in herd health restoration programs allows for the timely removal of infected animals and helps prevent further transmission to healthy livestock. The early identification of reactors through enhanced tuberculin sensitivity provides a critical advantage for controlling bTB in endemic herds, reducing economic losses, and facilitating the maintenance of TB-free herd status under national eradication programs.

#### One Health significance

The development of a phenol-free, formaldehyde-stabilized tuberculin formulation contributes directly to the One Health framework by strengthening the interface between animal, human, and environmental health. bTB, caused by *M. bovis*, is a recognized zoonosis that can infect humans through unpasteurized milk, direct animal contact, or aerosols. Enhancing diagnostic precision in livestock therefore reduces the zoonotic spillover risk and the potential for cross-species transmission. Improved detection of infected cattle supports national disease control programs, ensures the safety of animal-derived food products, and safeguards rural communities whose livelihoods depend on livestock. Consequently, the optimized formaldehyde-based tuberculin not only advances veterinary diagnostics but also aligns with global One Health objectives for the integrated management of TB across species.

## CONCLUSION

The present study demonstrated that substituting phenol with formaldehyde in tuberculin formulations maintains, and in certain concentrations, enhances the diagnostic activity of the allergen while improving its biosafety profile. Among the tested concentrations, 3% formaldehyde produced the highest mean biological activity (approximately 113% relative to the commercial PPD control) and exhibited the most stable immunological performance across guinea pigs, calves, and field-tested cattle. This concentration provided stronger DTH reactions, increased skin-fold thickness, and superior diagnostic sensitivity without inducing non-specific responses or local irritation. The experimental tuberculin stabilized with 3% formaldehyde achieved an AUC of 0.928, confirming excellent diagnostic accuracy and a higher rate of detection of *M. bovis*-infected animals compared with the standard phenol-stabilized PPD.

From a practical standpoint, this optimized tuberculin offers a safe, effective, and eco-friendly alternative for routine field diagnostics of bTB. The elimination of phenol as a preservative reduces toxicity risks to animals, laboratory personnel, and the environment, aligning with modern biosafety and sustainability standards. The enhanced sensitivity of the preparation enables the early identification and removal of reactors during herd screening, contributing to more effective eradication programs and minimizing economic losses in dairy and beef industries.

The strengths of this study lie in its multi-tiered design, spanning laboratory standardization, experimental validation on guinea pigs and calves, and confirmatory testing under field conditions in TB-affected herds. The integration of immunopathological and statistical analyses (including CI estimation, ANOVA, and ROC-based diagnostic validation) provides strong evidence supporting the functional reliability of the 3% formaldehyde formulation.

However, certain limitations must be acknowledged. The experimental sample size, particularly in calf trials, may have constrained the statistical power for distinguishing subtle differences between adjacent concentrations (e.g., 1% and 3%). Additionally, inter-individual variability in immune responsiveness and environmental factors such as stress, nutrition, and co-infections could influence skin-test reactivity. The study did not evaluate long-term stability, batch consistency, or storage temperature effects, which should be examined before large-scale implementation.

In terms of future scope, further research should focus on large-scale field validation across different breeds, age groups, and climatic regions to confirm diagnostic reproducibility under diverse conditions. Comparative studies using interferon-gamma release assays (IGRA) or PCR-based confirmation could help establish the tuberculin’s correlation with advanced molecular diagnostics. Exploring other mild stabilizers or adjuvants that complement formaldehyde’s preservative effect without altering antigenicity could also expand its practical utility. Additionally, establishing standardized international protocols for formaldehyde-based tuberculin production would strengthen cross-border disease control cooperation.

From a One Health perspective, the introduction of a phenol-free, formaldehyde-stabilized tuberculin contributes to reducing occupational exposure hazards, improving animal welfare, and limiting the zoonotic transmission potential of *M. bovis* to humans. By enhancing early detection at the livestock level, this innovation directly supports global TB control goals across animal and human health sectors.

In conclusion, tuberculin stabilized with 3% formaldehyde represents a scientifically validated and operationally feasible improvement over conventional formulations. It offers higher sensitivity, comparable specificity, better safety, and greater environmental compatibility. Its adoption in national TB surveillance and eradication programs could significantly strengthen diagnostic capacity and promote sustainable, One Health–aligned control of bTB.

## AUTHORS’ CONTRIBUTIONS

KT: Conceptualization, supervision, and coordination of research. AB: Development of the methodology, preparation and standardization of experimental allergens, and manuscript drafting. AO: Microbiological cultivation and identification of “*M. bovis*” and atypical mycobacteria, and contribution to the experimental design. SD: Animal experiments in calves, assessment of allergic skin reactions, and data collection. AT: Field trials on cattle, processing of diagnostic data, and statistical analysis support. RT: Preparation of figures and tables, data visualization, and manuscript revision. All authors have read and approved the final version of the manuscript.
